# Exponentially index modulated nanophotonic resonator for high-performance sensing applications

**DOI:** 10.1038/s41598-023-28235-6

**Published:** 2023-01-25

**Authors:** Diptimayee Dash, Jasmine Saini, Amit Kumar Goyal, Yehia Massoud

**Affiliations:** 1 Department of Electronics and Communication Engineering, Jaypee Institute of Information Technology, Noida, 201309 India; 2 Innovative Technologies Laboratories (ITL), King Abdullah University of Science and Technology (KAUST), Thuwal, 23955 Saudi Arabia

**Keywords:** Optical sensors, Sensors, Nanophotonics and plasmonics

## Abstract

In this manuscript, a novel photonic crystal resonator (PhCR) structure having an exponentially graded refractive index profile is proposed to regulate and alter the dispersion characteristics for the first time. The structure comprises silicon material, where porosity is deliberately introduced to modulate the refractive index profile locally. The structural parameters are optimized to have a resonant wavelength of 1550 nm. Further, the impact of various parameters like incidence angle, defect layer thickness, and analyte infiltration on device performance is evaluated. Finally, the sensing capability of the proposed structure is compared with the conventional step index-based devices. The proposed structure exhibits an average sensitivity of 54.16 nm/RIU and 500.12 nm/RIU for step index and exponentially graded index structures. This exhibits the generation of a lower energy resonating mode having 825% higher sensitivity than conventional resonator structures. Moreover, the graded index structures show a 45% higher field confinement than the conventional PhCR structure.

## Introduction

The development of bio-photonic sensors having a stable and fast response, along with low-level label-free detection capability, has been a recent research area globally^1,2^. Although the accuracy and precision of conventional analytical procedures are very high, but they are very expensive and consume a lot of analytes. Due to their increased specific surface area, little attenuation of light, ease of production, and characterization, periodic photonic crystals (PhC) resonating nanostructures have recently attracted a lot of attention from the research community^3–5^. Resonating one-dimensional (1D) PhC devices have seen a significant increase in demand over the past few years in a range of sensing applications, including liquid sensing, gas sensing, and biomedical diagnostics^6,7^. Most PhC sensors evaluate various optical properties, such as transmittance, reflectance, bandgap, and mode profile, based on the variation in the refractive index of analytes^8–10^. These structures are constructed from two or more materials with different refractive indices arranged in a repetitive pattern. In the conventional PhC resonator structures, the structural parameters are generally optimized to obtain a desired resonating wavelength, and defect layer width is optimized to improve the sensitivity^11,12^. However, considering the gradient refractive index (GRI) distribution, an additional degree of design freedom can be attained, which further help to improve the device performance^13,14^.

The graded PhC devices are designed by modulating the structural and material parameters like lattice constant, optical length, and filling factor^15^. The graded profile can be extended to control and manipulate light propagation within the Nanophotonics device^16^. This decreases the interface-assisted losses and provides an efficient way to develop a graded 1D-PhC structure having improved performance characteristics than conventional 1D-PhC structures. The optical length can be modulated by gradually changing refractive index profiles along the width of the layers. Centeno et al. proposed the first graded PhC structure with lattice constant modulation^17,18^. Later, it was looked into for several uses, including couplers^19^, optical lenses^20^, mode converters^21,22^, and optical multiplexers^23,24^. Major applications of optical length modulated grade PhC structure include super bending, super collimation, high-efficiency couplings, super lensing, beam aperture modifier, and deflector^25,26^. Refractive index modulation is another widely explored method to study the band-gap features of the PhC structures. Recently in 2023, Savotchenko considered parabolic and linear refractive index profile-based structures to study the new type of surface wave propagation and described its wave-guiding properties^27,28^. The refractive index modulation-assisted 1D-PhC designs are widely explored to study the bandgap properties. The linear GRI configuration is used to study the optical transmittance property of the 1D-PhC structure^29^. The band gap properties of other GRI configurations (such as exponential, logarithms, and hyperbolic) based on 1D-PhC structures have also been explored^30,31,32^. However, to our knowledge, work has yet to be reported in the literature about exponential GRI-based PhC resonator configurations for sensing applications.

This research proposes a novel PhC cavity structure with a graded refractive index that can regulate and alter light propagation. The structure's design comprises a porous bilayer 1D-PhC structure made of silicon and porous silicon, in which the high index layer's refractive index is exponentially modulated along its width. The inclusion of porosity makes it possible to achieve a very large contrast in refractive index within the same material, which results in essentially minimal interface-induced losses. Additionally, it makes it easier to adjust the mode dispersion properties and offers design scalability at any wavelength range specified by the user. Moreover, it enables analyte infiltration for sensing applications simpler. For both step index and graded index PhC cavity structures, the effect of various parameters, such as incidence angle, defect layer thickness, and analyte infiltration, on device performance are investigated. Finally, the proposed structure's sensing capability is compared with conventional step index-based devices. In contrast to the conventional resonator structures, this shows the generation of a lower energy resonant mode with 825% higher sensitivity. Moreover, the graded index structures show a 45% higher field confinement compared to the conventional PhCR structure.

The paper is organized into three major sections. The proposed structure and used calculation method, which is based on the transfer matrix method (TMM), is discussed in the “Theoretical model and analysis” section. The reflection coefficient and dispersion relation are determined in this section. The discussion of this reflection spectra for the Psi-based periodic multilayer structures containing exponentially graded index material is summarized in “Results and discussions”. Finally, the last section is devoted to “Conclusions”.

## Theoretical model and analysis

**Figure 1:**
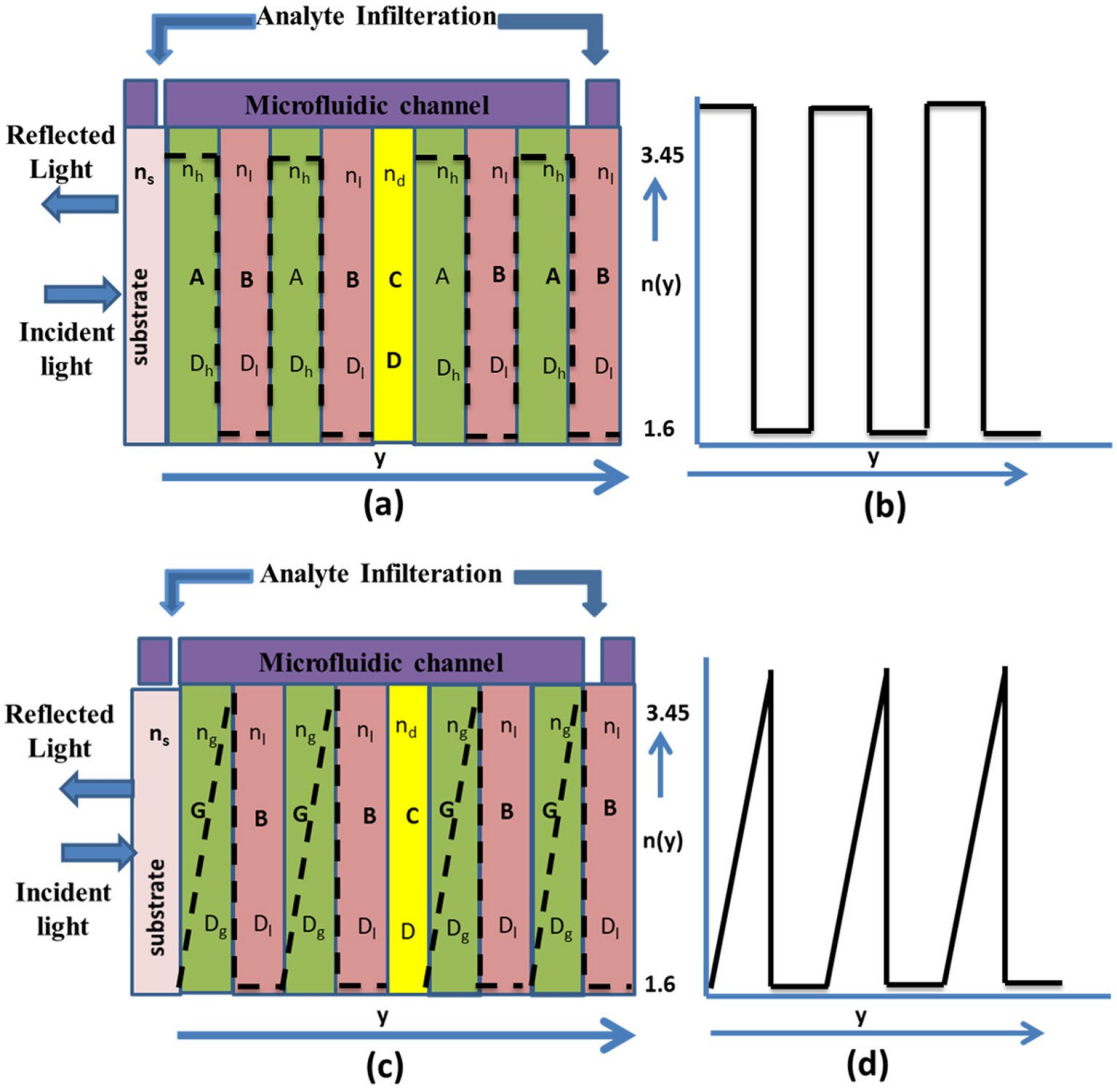
Schematic representation of proposed structure. (**a**) Step index photonic crystal resonator, (**b**) corresponding refractive index variation, (**c**) Exponentially Graded index multilayer resonator structure, and (**d**) corresponding refractive index variation.

A bilayer PhC structure is designed by considering alternate layers of high index material ‘A’ and low index material ‘B’ on BK7 substrate and is represented in Fig. 1. A homogeneous layer of 1D-PhC with the defect layer ‘C’ as [(A/B)^3^/C/(A/B)^3^] makes up the conventional step index photonic crystal resonator (SI-PhCR) as shown in Fig. 1a and corresponding refractive index profile is represented in Fig. 1b. The physical thicknesses of layers are calculated by considering quarter wavelength Bragg stack having n × d = λo/4, where ‘n’ is the refractive index, ‘d’ is the physical thickness and λo is the central operating wavelength (here, 1550 nm)^33,34^. In the analysis, porous Si is referred to as material ‘B’ and has a refractive index (n_l_) of 1.6 (Si with 80% porosity) and a thickness (D_l_) of 242 nm, compared to Si, which has a refractive index (n_h_) of 3.45 (Si with 0% porosity) and a thickness (D_h_) of 112 nm.

The structure of an exponentially graded index resonator made up of two different types of dielectric layers is shown in Fig. 1c, and the corresponding refractive index profile is mentioned in Fig. 1d. The high index layer has an exponentially varying refractive index profile along the layer thickness, and the low index layer is considered a homogeneous material with a constant refractive index. The defect layer of refractive index (n_d_) and width (D) is represented by layer ‘C’. The refractive index of the exponentially graded index dielectric layer varies from 1.6 to 3.45, which is within the range of the refractive index of most dielectric materials. The addition of porosity makes it possible to realize the grading structure^35,36^.

1 $$P=\frac{({n}_{p}^{2}-{n}_{dense}^{2})({n}_{a}^{2}-{2n}_{dense}^{2})}{({n}_{p}^{2}+2{n}_{dense}^{2})({n}_{a}^{2}-{n}_{dense}^{2})}$$

where ${n}_{p}$, ${n}_{dense}$ and ${n}_{a}$ are the Refractive index of porous material, dense material, and air/analytes, respectively. The Sellmeier approximation is used to compute the refractive index of bulk silicon, while Eq. (1) is used to calculate porous silicon’s refractive index, those are mentioned in Table 1.

**Table 1:** Effective refractive index of silicon with various porosity percentages.

Porosity (%)	Si (refractive index)
0	3.45
10	3.2248
20	3.0029
30	2.7826
40	2.5619
50	2.3386
60	2.1097
70	1.8712
80	1.6166
90	1.3346

The exponentially graded index photonic crystal resonator (EG-PhCR) is a 1D-PhCR with a graded refractive index profile in the configuration [(G/B)^5^/C/(G/B)^5^], where ‘G’ refers to the material with the graded refractive index. With increasing layer width (D_g_), the refractive index distributions in graded layer ‘G’ changes exponentially. According to Fig. 1c,d, this varies from 1.6 to 3.45, while layer ‘B’ is considered to be a porous layer with a low refractive index (n_l_) of 1.6 (Si with 80% porosity). The graded layer's width is calculated as D_g_ = λo/(4*n_avg_), where ‘n_avg_’ is the average of the layer's low and high refractive indices. This gives the defect layer thickness (D_g_) of around 153 nm. The exponential index variation from initial (y = 0) to final (y = d_A_) in Graded material can be calculated using Eq. (2)^31,37^.

2 $${n}_{E}\left(y\right)={n}_{i}exp(\beta y)$$

where $\beta =\left(\frac{1}{t}\right).log(\frac{{n}_{i}}{{n}_{f}})$ is the exponential grading parameter, and subscript ‘E’ represents the exponentially graded index layer. The parameters ‘n_i_’ and ‘n_f_’ are the initial and final refractive indices of the exponentially graded layer. Here, ‘i’ is the initial, and ‘f’ is the final position along the layer’s width. The distribution of the electric field along the plane perpendicular to the exponentially grading layer's surface is given by Eq. (3)

3 $${E}_{E\left(y\right) }={A}_{E}.{J}_{0}\frac{{\xi }_{E}}{\beta }+{B}_{E}.{Y}_{0}\frac{{\xi }_{E}}{\beta }$$

${A}_{E}$ and ${B}_{E}$ are the constants and ${J}_{0}$ and ${Y}_{0}$ are the zeroth order Bessel functions. At a 90° incidence angle, the wave propagation vector of the exponentially graded index layer is written as ${\xi }_{E}={n}_{E}\left(y\right).\omega /c$, Where ‘$\omega$’ is the angular frequency and ‘$c$’ is the speed of light^31^. The TMM (Transfer Matrix Method) is highly suited to be considered for 1D resonator-based graded (exponentially variable) and non-graded structures to assess the reflection spectrum of the structure. The transfer matrix of a particular nth layer is expressed as in Eq. (4).

4 $${M}_{n}=\left[\begin{array}{cc}\mathrm{cos}{\alpha }_{n}& \frac{{jsin\alpha }_{n}}{{p}_{n}}\\ -j{p}_{n}{sin\alpha }_{n}& \mathrm{cos}{\alpha }_{n}\end{array}\right]$$

where ${\alpha }_{n }= \frac{2\prod}{\lambda {n}_{n}{d}_{n}coscos {\theta }_{n}}$ and p_n_ = ${n}_{n}cos\,cos {\theta }_{n}$ for TE mode, ${n}_{n}, {d}_{n}\, and \,{\theta }_{n}$ are the refractive index, thickness and propagation angle for nth layer and ‘$\lambda$’ is the resonant wavelength. The resonant wavelength taken into consideration is 1550 nm. For the proposed multilayer structure, the final characteristic matrix can be found by multiplying each matrix for individual layers and is represented by Eq. (5).

5 $$M = \prod\nolimits_{n = 1}^{N} {M_{n} = \left[ {\begin{array}{*{20}c} {M_{11} } & {M_{12} } \\ {M_{21} } & {M_{22} } \\ \end{array} } \right]}$$

where ‘N’ is the number of periods. The transmission coefficient for the multilayer proposed structure is then evaluated and is given by Eq. (6).

6 $$t=\frac{2 \mathrm\,{cos}\,\theta}{{(M}_{11}+{M}_{12 }\mathrm{cos}\theta)+{(M}_{21}+{M}_{22 }\mathrm{cos}\theta)}$$

Finally, the transmittance of the structure can be written by using the above reflection coefficient as $T={\left|t\right|}^{2}$ having R + T = 1. Thus, the reflectance of the structure can be found to be (1 − T). Therefore, using these equations, the optical reflections, band structure, resonant wavelength, and electric field profile for both SI-PhCR and EG-PhCR-based 1D photonic structures are calculated.

## Results and discussions

**Figure 2:**
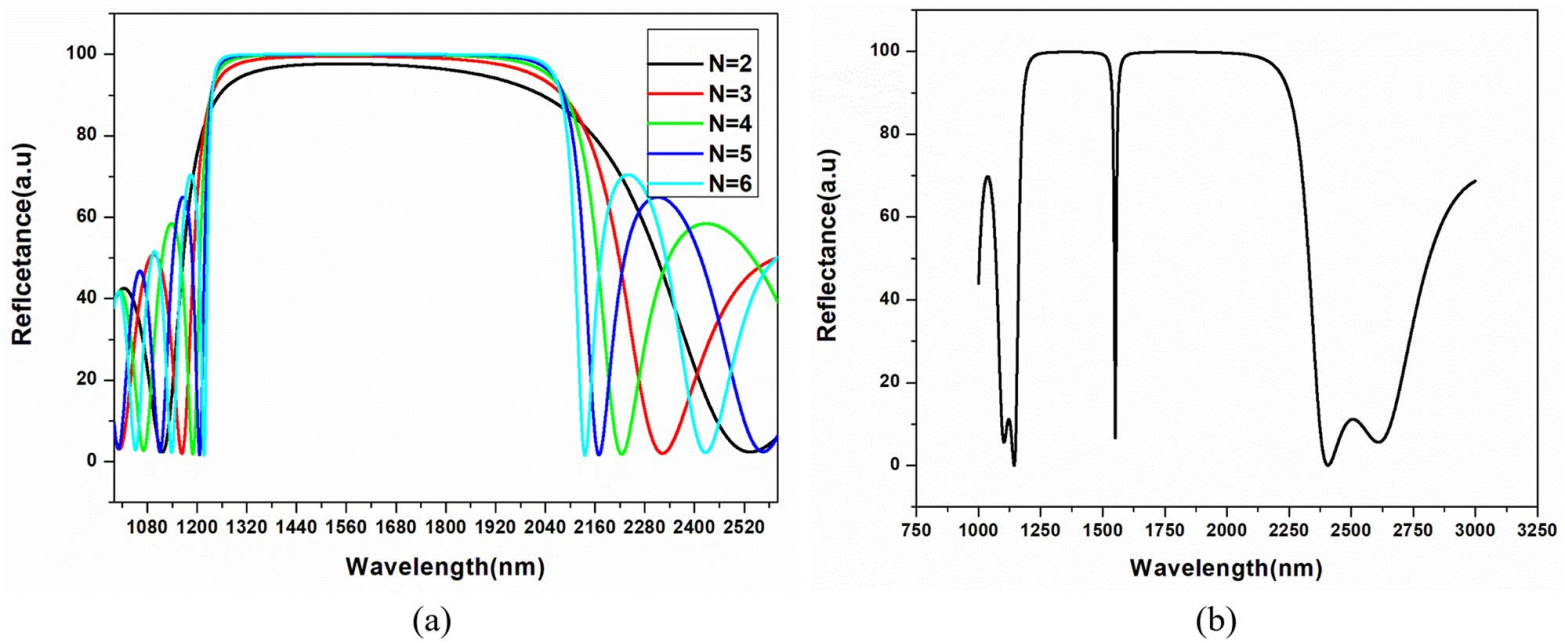
Reflection spectra at normal incidence angle for (**a**) 1D-PhC structure (glass/(AB)^N^/air), and (**b**) 1D-PhCR structure (glass/(AB)^3^/C/(AB)^3^/air) having D = D_h_ and n_d_ = n_h_.

**Figure 3:**
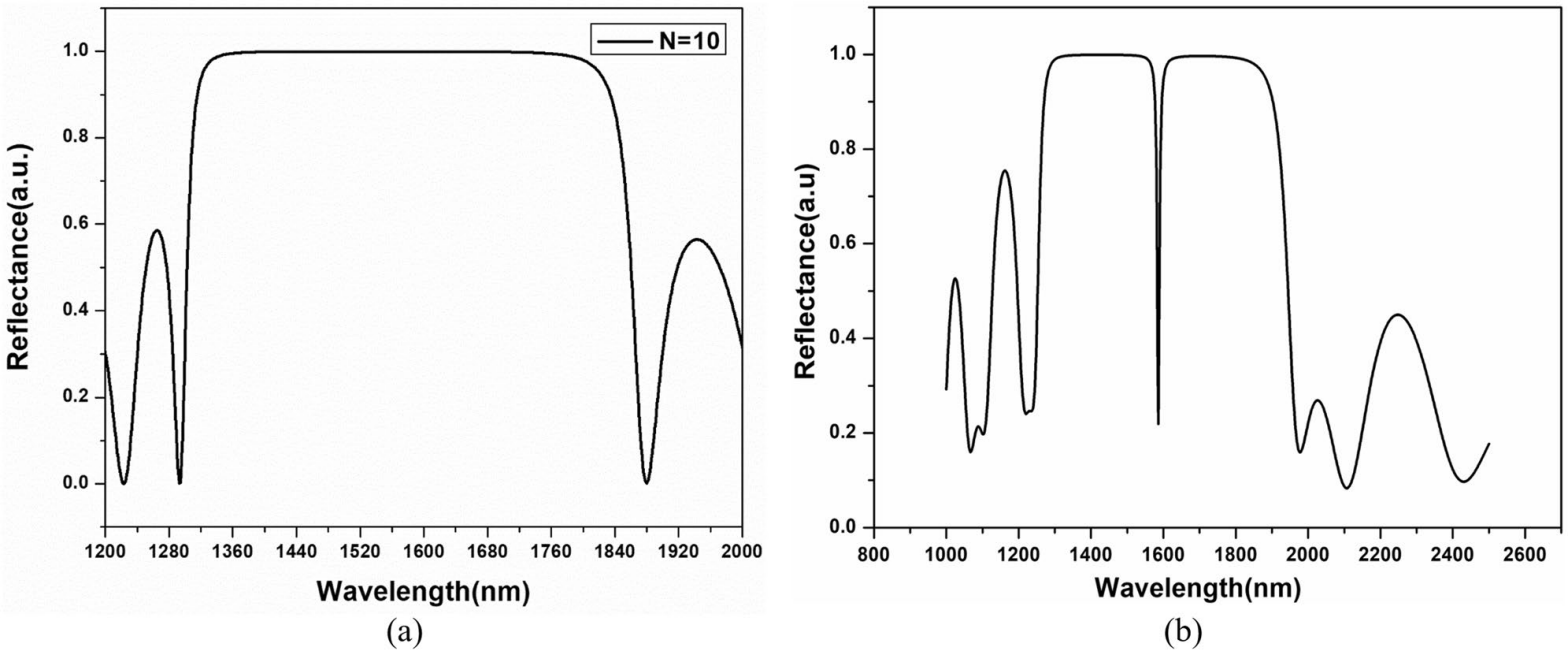
Reflection spectra at normal incidence angle for (**a**) 1D-PhC structure (glass/(AB)^N^/air), and (**b**) 1D-PhCR structure (glass/(GB)^5^/C/(GB)^5^/air) having D = D_g_ and n_d_ = n_g_.

The analysis is carried out using the transfer matrix method (TMM) and the finite element method (FEM). The TMM calculates the structure's bandgap and reflection/transmission coefficient, whereas the finite element method (FEM) is used to validate the structure performance by analyzing the interface electric field. Initially, a 1D-PhC multilayer structure without any defect is investigated. The reflection spectrum curve formed using FEM for SI-PhCR is shown in Fig. 2. The structure shows a photonic bandgap formation in the near-IR region (1250–2100 nm), where sharper photonic bandgap edges can be obtained by increasing the periods (‘N’). The reflection spectrum curve for GI-PhCR is shown in Fig. 3. The bandgap for GI-PhCR structure shows a photonic bandgap formation (1280–1880 nm). The addition of a defect layer into the middle of the multilayer structure localizes the defect modes. This results in a reflection dip at a central wavelength of 1550 nm, as shown in Fig. 2b for the SI-PhCR structure. Whereas the reflection dip at a central wavelength of 1585 nm is obtained for GI-PhCR structure, as shown in Fig. 3b. This resonating mode is very sensitive to a small perturbation and can be tuned by varying the optical length of the defect region. Moreover, the incidence angular variation also affects the resonating mode properties. Thus, the performance assessment of the proposed EG-PhCR structure is carried out by considering variations in the defect layer's physical thickness and refractive index. The defect layer is infiltrated with various analytes, and the corresponding change in resonance wavelength is recorded.

Considering this, several performance assessment factors, including Sensitivity, FOM, and Detection Limit (DL), are calculated to accurately assess the usefulness of the proposed sensor. The sensitivity (S) of the sensor is measured as the ratio of change in position of defect mode wavelength ($\Delta {\lambda }_{d}$) in the defect layer to a corresponding change in refractive index $(\Delta {n}_{d })$ of analyte and can be calculated by $S=\frac{\Delta {\lambda }_{d}}{\Delta {n}_{d}}$. The FOM represents the capacity to detect the smallest variation in defect mode resonance wavelength, which can be calculated by $FOM=\frac{S}{\Delta {\lambda }_{1/2}}$, where $\Delta {\lambda }_{1/2}$ is the spectral half width of the reflection dip. The detection limit refers to a sensor's capacity to recognise very small refractive index contrasts of the analyte and can be measured by $DL=\frac{R}{S}$.

### Impact of variation in incidence angle and defect layer thickness

**Figure 4:**
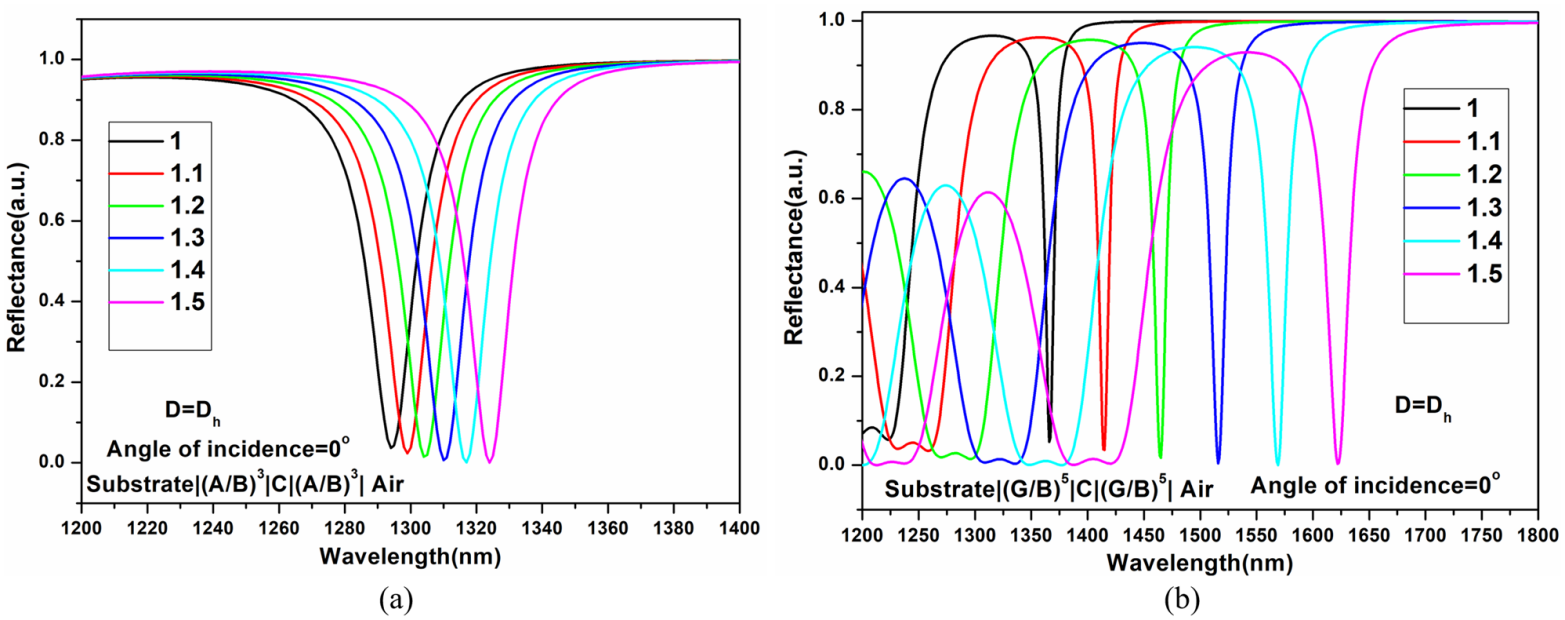
Impact of analyte infiltration in the structure of defect layer thickness D = D_h_ for (**a**) SI-PhCR structure and (**b**) EG-PhCR structure.

First, the effect of different defect layer thicknesses on resonating wavelengths is examined for varied infiltrating analyte concentrations. The addition of porosity makes it easier for analytes to penetrate the layers. The effective refractive index changes, as a result, shifting the resonance wavelength of the resonator in the reflection spectrum, as seen in Fig. 4. Analytes with refractive indices ranging from 1.0 to 1.5 with steps of 0.1 are introduced into the defect layer, and the resulting reflection spectrum is analyzed. The low index layer's effective refractive index (RI) is maintained constant at 1.6166, 1.6744, 1.7354, 1.7991, 1.8653, and 1.9337 for corresponding refractive index variations of 1.0, 1.1, 1.2, 1.3, 1.4, and 1.5, respectively, while the effective refractive index of the graded layer changes exponentially from 1.6166 to 3.45.

According to the analysis, using the same materials and constant parameters with the EG-PhCR structure results in the formation of lower energy resonant modes. Additionally, the resonant energy decreases as the defect layer's effective index rises. The study is further developed to compare the SI-PhCR and EG-PhCR sensing capacities for increasing defect layer thicknesses at oblique incidence angles. As indicated in the supplementary file attached, the effect of increasing defect layer thickness D (D_h_ to 3D_h_) on the sensitivity of the SI-PhCR and EG-PhCR structure is also analyzed (0°, 20°, and 40° incidence angle). Supplementary Fig. S1 depicts the comparison of varying defect layer thickness ‘D’ (2D_h_ & 3D_h_) on sensitivity for the structure “Substrate**/**(A**/**B)^3^**/**C**/**(A**/**B)^3^**/**Air” and “Substrate**/**(G**/**B)^5^**/**C **/**(G**/**B)^5^**/**Air” with keeping the angle of incidence to be constant at 0°. Supplementary Fig. S2 shows the comparison of varying defect layer thickness D (D_h_ to 3D_h_) on sensitivity for the structure “Substrate**/**(A**/**B)^3^**/**C**/**(A**/**B)^3^**/**Air” and “Substrate**/**(G**/**B)^5^**/**C**/**(G**/**B)^5^**/**Air” with keeping the angle of incidence to be constant at 20°. Similarly, the comparison of varying defect layer thickness D (D_h_ to 3D_h_) on sensitivity for the structure “Substrate**/**(A**/**B)^3^**/**C**/**(A**/**B)^3^**/**Air” and “Substrate**/**(G**/**B)^5^**/**C**/**(G**/**B)^5^**/**Air” with keeping the angle of incidence to be constant at 40° has been shown in Supplementary Fig. S3 and results are summarized in Tables 2, 3, and 4.

**Table 2:** Sensitivity comparison of SI-PhCR and EG-PhCR Structure with different defect layer thickness at a 0° incidence angle.

Analyte refractive index	Step index PhC structure sensitivity (nm/RIU)			Graded index PhC structure sensitivity (nm/RIU)		
	D = D_h_	D = 2D_h_	D = 3D_h_	D = D_h_	D = 2D_h_	D = 3D_h_
1.1	50	150	300	490	570	660
1.2	50	165	320	495	575	695
1.3	53.3	170	333.3	496.6	593.3	673
1.4	57.5	180	340	505	597.5	665
1.5	60	188	340	514	586	652

**Table 3:** Sensitivity comparison of SI-PhCR and EG-PhCR Structure with different defect layer thickness at a 20° incidence angle.

Analyte refractive index	Step index layer sensitivity (nm/RIU)			Graded index layer sensitivity (nm/RIU)		
	D = D_h_	D = 2D_h_	D = 3D_h_	D = D_h_	D = 2D_h_	D = 3D_h_
1.1	50	170	340	480	630	790
1.2	60	175	350	525	635	790
1.3	56.66	186	333.3	526	646	770
1.4	62.5	197.5	370	537.5	642.5	752.5
1.5	66	208	382	540	642	740

**Table 4:** Sensitivity comparison of SI-PhCR and EG-PhCR Structure with different defect layer thickness at a 40° incidence angle.

Analyte refractive index	Step index layer sensitivity (nm/RIU)			Graded index layer sensitivity (nm/RIU)		
	D = D_h_	D = 2D_h_	D = 3D_h_	D = D_h_	D = 2D_h_	D = 3D_h_
1.1	60	170	320	610	800	980
1.2	60	190	365	625	805	1010
1.3	66.6	216	413.3	630	806.6	1020
1.4	70	227.5	447.5	637.5	807.5	1005
1.5	76	246	472	640	802	986

**Figure 5:**
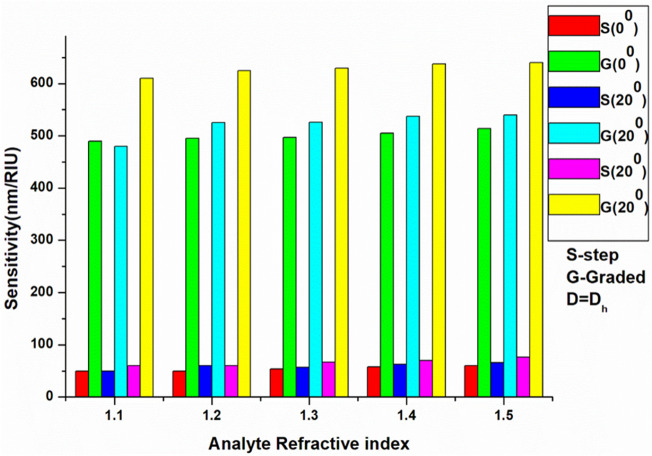
Sensitivity comparison of step index and graded index structure with different incidence angle and defect layer thickness D = D_h._

**Figure 6:**
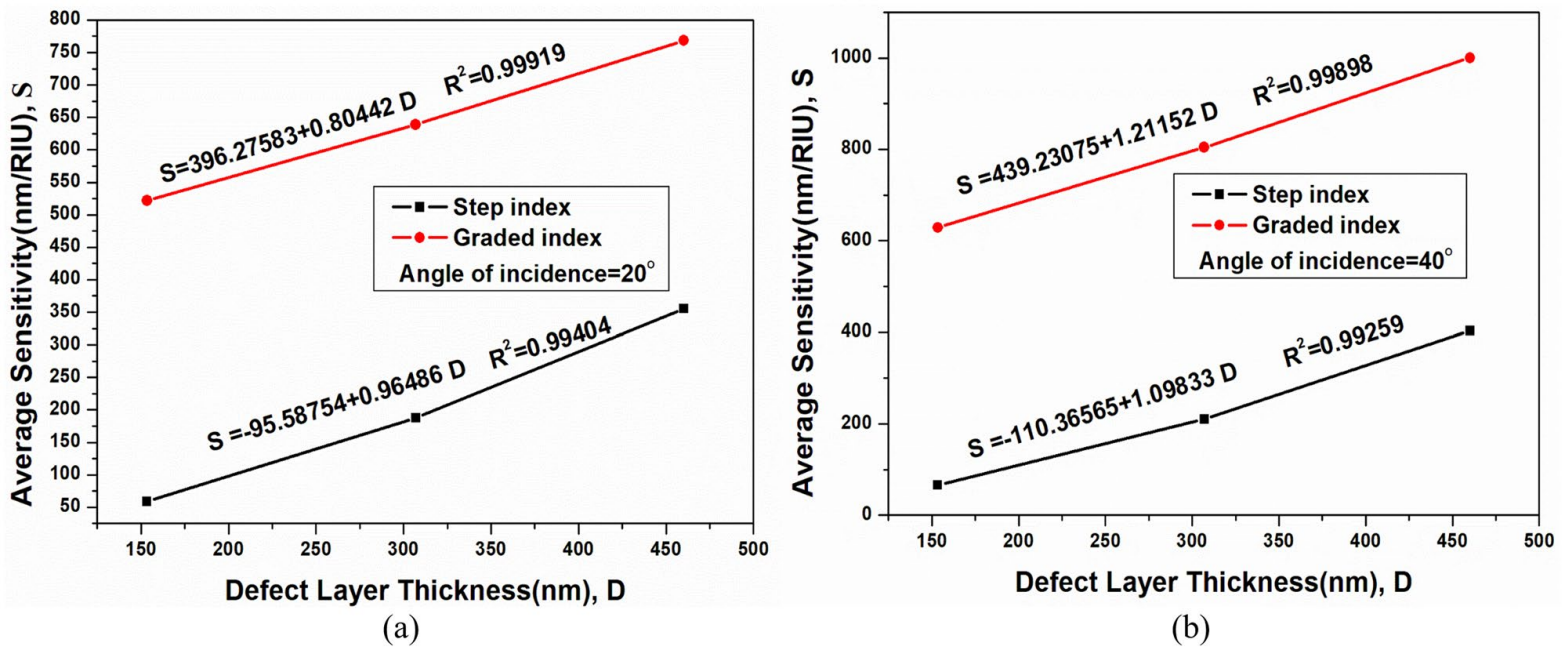
Sensitivity comparison of step index and graded index structure with different defect layer thickness with (**a**)incidence angle = 20° (**b**) incidence angle = 40°.

As shown in Tables 2, 3, and 4, the sensitivity of the SI-PhCR and EG-PhCR structures are compared for angles of incidence of 0°, 20°, and 40°, as well as for changing the thickness of the defect layer from D_h_ to 3D_h_. Bar chart representation of the sensitivity variation with a constant defect layer thickness (D_h_) and variable incidence angle is represented in Fig. 5. Figure 6 summarizes the average sensitivity comparative analysis of SI-PhCR and EG-PhCR structure for different defect layer thicknesses at a constant incidence angles of 20° and 40°.

The sensing comparison for a 20° incidence angle is shown in Fig. 6a. The response demonstrates a linear sensing variation for both SI-PhCR and EG-PhCR structures. The average sensitivity can be calculated by Eqs. (7) (for SI-PhCR) and 8 (for EG-PhCR), which are obtained by linear fitting the curve.

7 $$\mathrm{S }=-95.58754+0.96486\mathrm{ D}$$

8 $$\mathrm{S}=396.27583+0.80442\mathrm{ D}$$

From linear fit, we obtain the slope of $\partial S/\partial D=0.96486$ for SI-PhCR structure and $\partial S/\partial D=0.80442$ for EG-PhCR structure along with the coefficients of determination of R^2^ of around 0.99919 and 0.99404, respectively. Similarly, Fig. 6b represents the linear fit curve for a 40° incidence angle with a slope of $\partial S/\partial D=1.09833$ for SI-PhCR structure and $\partial S/\partial D=1.21152$ for EG-PhCR structure along with the coefficients of determination of R^2^ of around 0.99898 and 0.99259, respectively. The average sensitivity response for the same is presented in Eq. (9) for SI-PhCR structure and Eq. (10) for EG-PhCR structure.

9 $$\mathrm{S }= -110.36565+1.09833\mathrm{ D}$$

10 $$\mathrm{S }= 439.23075+1.21152\mathrm{ D}$$

From Eqs. (7–10), it can be concluded that the average sensitivity (S) and defect layer thickness (D) (at a fixed incidence angle) show a good linear relation. It is clear from the graph that infiltration of the analyte at the center of the resonator exhibits an excellent linear dependency for the smaller refractive index variation. For defect layer thickness D_h_, higher energy bands are excited within the resonator. As we increase defect layer thickness from 2 to 3D_h_, lower energy bands are excited. If defect layer thickness is increased beyond 3D_h_, leaky mode or unconfined modes are excited, which are outside our desired bandgap. Therefore, the defect layer thickness is being considered up to 3D_h_ only.

Based on the above observation, the average sensitivity of the SI-PhCR structure is 54.16 nm/RIU for a defect layer thickness of D, while the average sensitivity of the EG-PhCR structure is 500.12 nm/RIU for an incidence angle of 0°. Similar to this, the EG-PhCR structure has an average sensitivity of 1000.2 nm/RIU, and the SI-PhCR structure has an average sensitivity of 403.06 nm/RIU with increasing defect layer thickness from D_h_ to 3D_h_ and at a 40° angle of incidence. The sensitivity of the EG-PhCR structure is thus 825% higher than that of a traditional SI-PhCR device. EG-PhCR structure exhibits FOM of about 1000 RIU^−1^, and a Limit of detection of about 2.66 × 10^–3^. The suggested structure has better performance and relatively higher sensitivity for detecting analytes at various concentrations. Major applications and previously reported designs are covered in the subsequent section.

### Surface electric field profile

**Figure 7:**
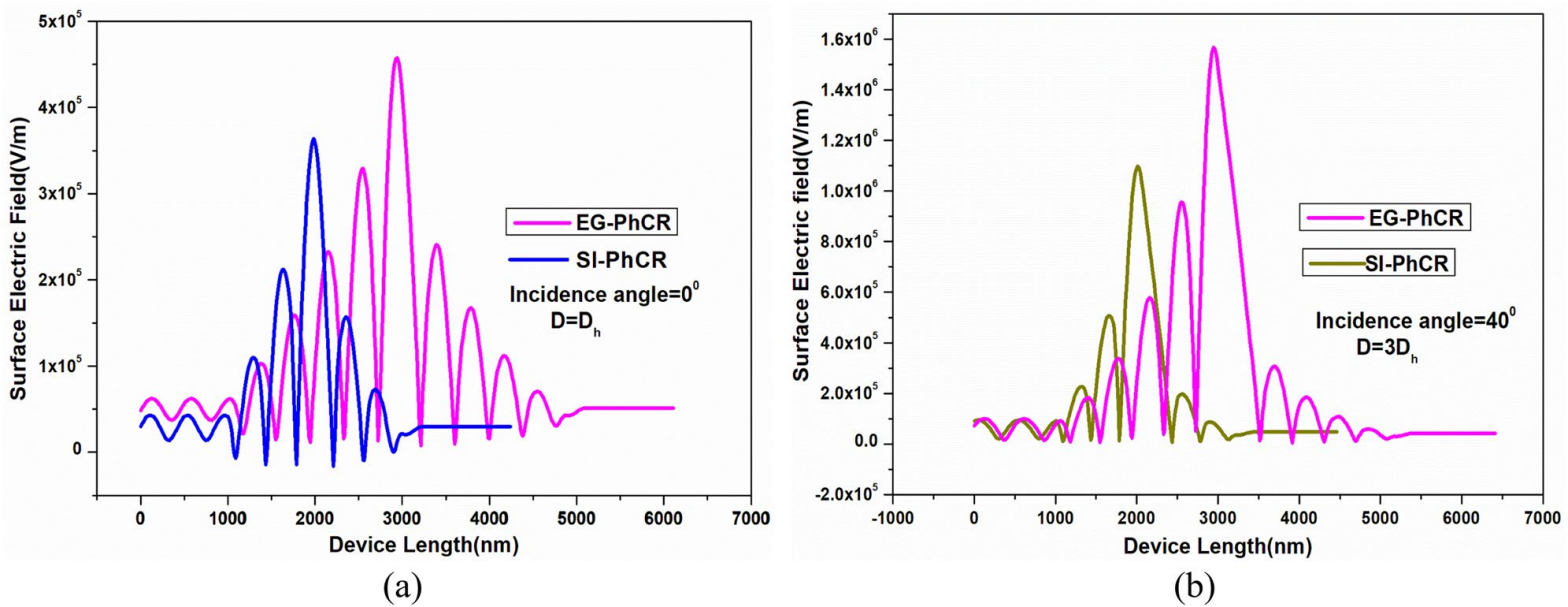
Surface electric field profile comparison of SI-PhCR structure and EG-PhCR structure with defect RI of ‘1’ at (**a**) 0° incidence angle and D_h_ defect layer thickness and (**b**) 40° incidence angle and 3D_h_ defect layer thickness.

The proposed novel Exponentially Graded structure possesses relatively much higher sensitivity than the Step index structure, which can be verified by analyzing its spectral field distribution, as shown in Fig. 7. Figure 7a represents the surface electric field intensity distribution for the considered structure having an incidence angle of 0° and Defect layer thickness of D_h_. The obtained surface electric field intensity in PhC resonator is 3.6 × 10^5^ V/m for the SI-PhCR structure, whereas it increases to 4.5** × **10^5^ V/m for EG-PhCR structure. Similarly, increasing the defect layer thickness to 3D_h_ at a 40° incidence angle, the obtained surface electric field is around 1.1 × 10^6^ V/m for the SI-PhCR structure and 1.6** × **10^6^ V/m for the EG-PhCR structure, which is shown in Fig. 7b. This leads to about a 45% increase in the electric field intensity in the EG-PhCR structure as compared to the SI-PhCR structure.

Finally, the structure performance is compared with recently reported designs and is given in Table 5. In comparison to recently reported works, our proposed GI-PhCR structure demonstrates substantially superior performance and relatively greater sensitivity for detecting different analyte concentrations. The proposed graded index RI-based sensor with high sensitivity has a great potential for gas sensing (RI of 1–1.3 ranges), liquid sensing, and biochemical (as major RI of biological components ranges between 1.3 and 1.5) sensing applications.

**Table 5:** Sensitivity comparison of the proposed sensor with previously reported sensors.

Year	Sensitivity (nm/RIU)	FOM (RIU^−1^)	LOD	Application	References
2023	290	1074	0.0668	Cancer cells sensing	^38^
2022	612.3	199	–	Salinity sensor	^39^
2022	203.09	–	0.0093	Dengue virus detection sensor	^40^
2022	144.369	–	–	Detection of water concentration in ethanol solution	^41^
Proposed work	1000	1000	0.00266	Various applications	–

## Conclusion

In the present work, a “Substrate/[GB]^5^/C/[GB]^5^/air” configuration of an exponentially graded photonic crystal resonator structure is investigated for photonic sensing application. To get the best results, structural factors such as layer count, defect layer thickness, grading profile, angle of incidence, and porosity values are optimized. The suggested GI-PhCR exhibits an 825% better sensitivity than the traditional SI-PhCR at normal incidence for the D_h_ cavity thickness. Further, the sensitivity can be enhanced to 1000 nm/RIU for the cavity thickness of 3D_h_ of the proposed Exponentially Graded design at an angle of incidence of 40°. The acquired sensitivity is sufficiently high to identify analytes of very small concentrations. Therefore, our proposed GI-PhCR structure can be used to design various photonic sensors with high sensitivity having the ability to control and manipulate light efficiently.

## Supplementary Information

Supplementary Information.

## Data Availability

Data underlying the results presented in this paper are not publicly available at this time but may be obtained from the authors (A.K.G. and Y.M.) upon reasonable request.
