# Supplementary material for: Exponentially index modulated nanophotonic resonator for high-performance sensing applications
Source: Sci Rep. 2023 Jan 25;13:1431. doi: 10.1038/s41598-023-28235-6 (PMC9877018; doi:10.1038/s41598-023-28235-6)
Supplement: Supplementary file 1 — Supplementary Information. [file 41598_2023_28235_MOESM1_ESM.pdf]

Exponentially Index Modulated Nanophotonic Resonator for High-performance Sensing Applications

Diptimayee Dash[1], Jasmine Saini[1], Amit Kumar Goyal[2] and Yehia Massoud[2,*]

[1]Department of Electronics and Communication Engineering, Jaypee Institute of Information Technology, Noida, India-201309

[2] Innovative Technologies Laboratories (ITL), King Abdullah University of Science and Technology (KAUST), Thuwal, Saudi Arabia-23955

**Corresponding Author: yehia.massoud@kaust.edu.sa*

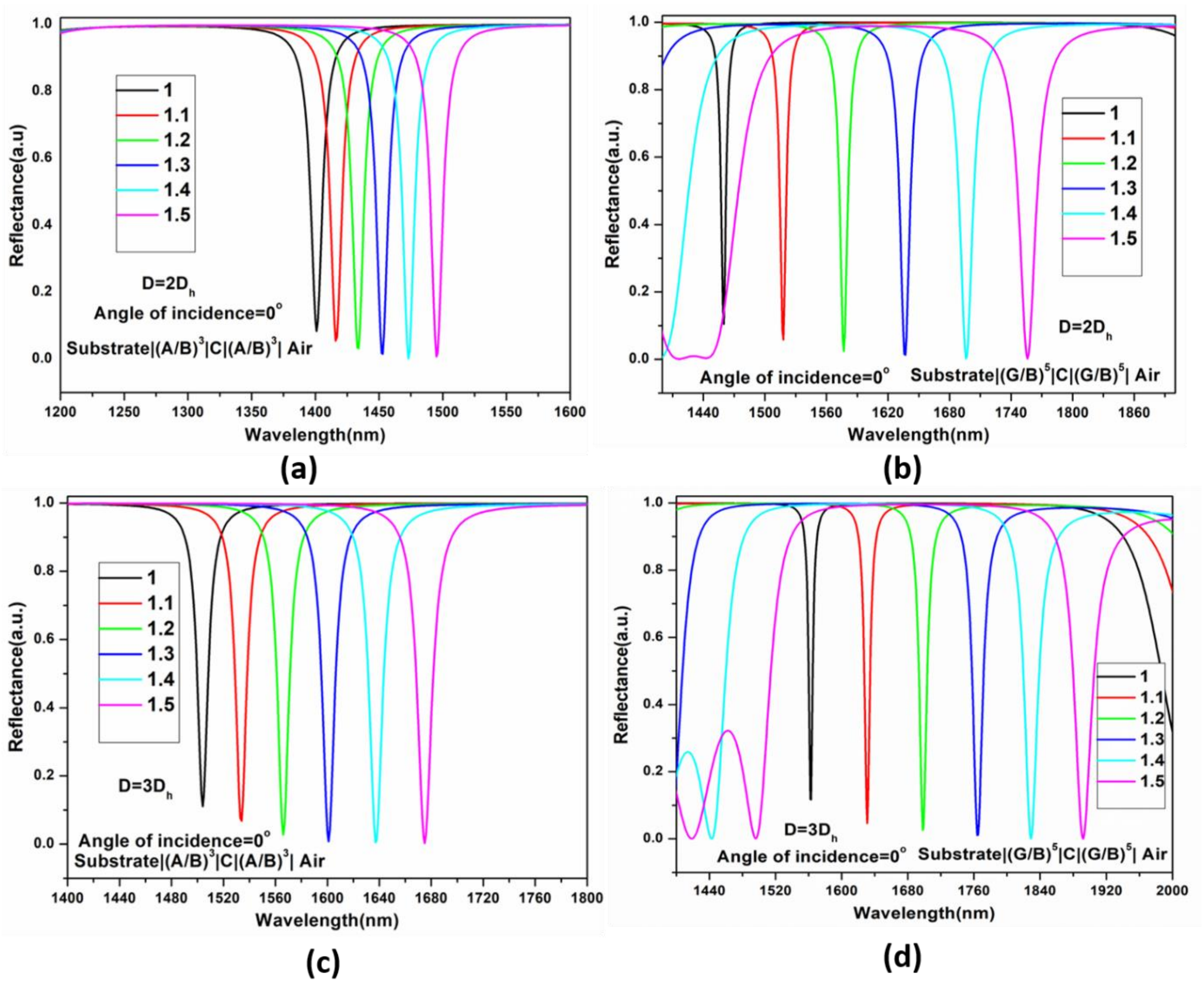

Fig. S1: Effect of varying defect layer thicknesses on sensitivity for the structure **Substrate/(A/B)^3 /C /(A/B)^3 /Air**, (a) D = 2D$_h$, (c) D = 3D$_h$ And for **Substrate/(G/B)^5 /C /(G/B)^5 /Air** (b) D = 2D$_h$, and (d) D = 3D$_h$. With incidence angle 0 degrees

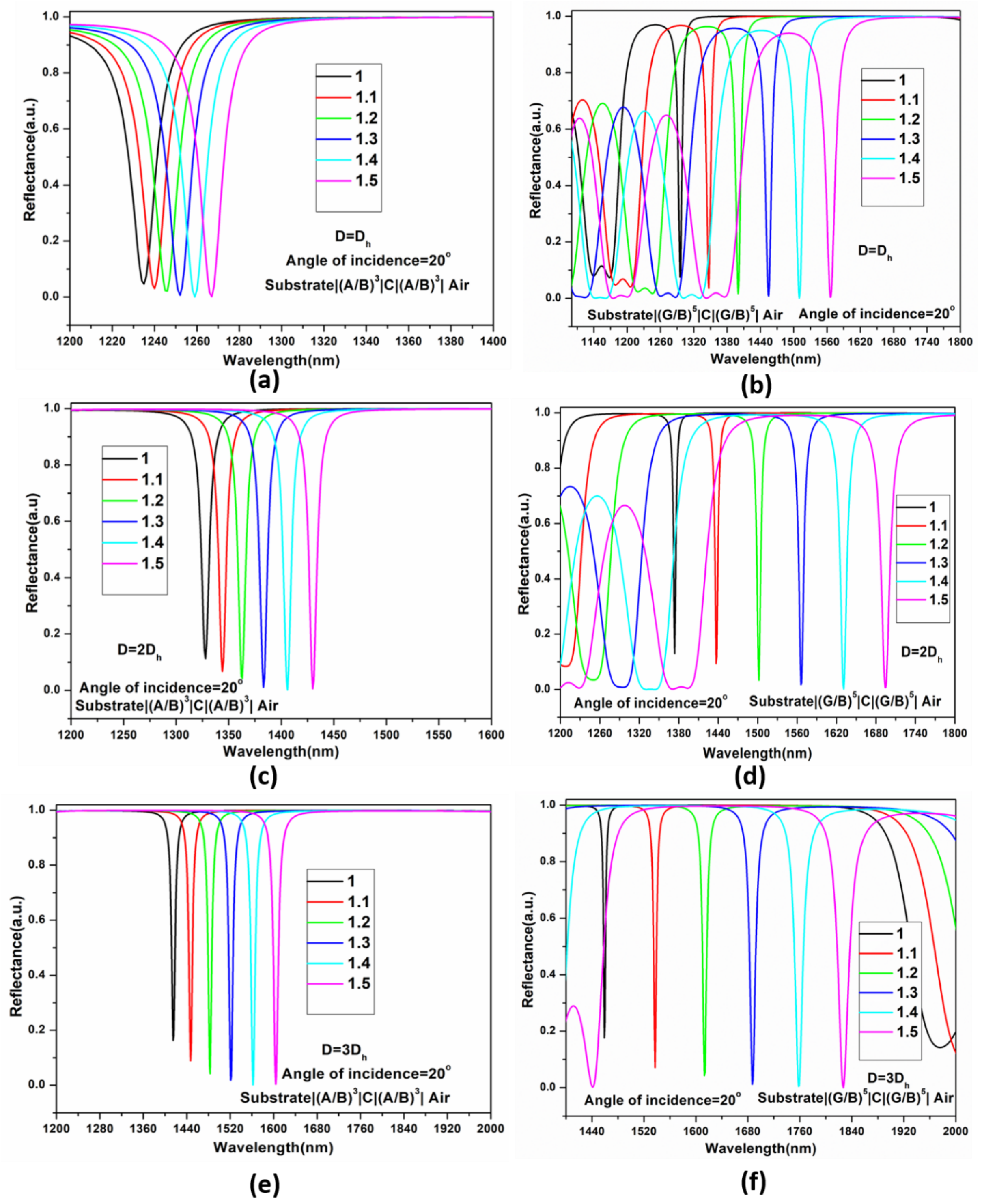

Fig. S2: Effect of varying defect layer thicknesses on sensitivity for the structure **Substrate/(A/B)^3 /C /(A/B)^3 /Air**, (a) D = D_h, (c) D = $2D_h$, (e) D =$3D_h$ And for **Substrate/(G/B)^5 /C /(G/B)^5 /Air** (b) D = D_h, (d) D =$2D_h$, and (f) D = $3D_h$. With incidence angle 20 degrees

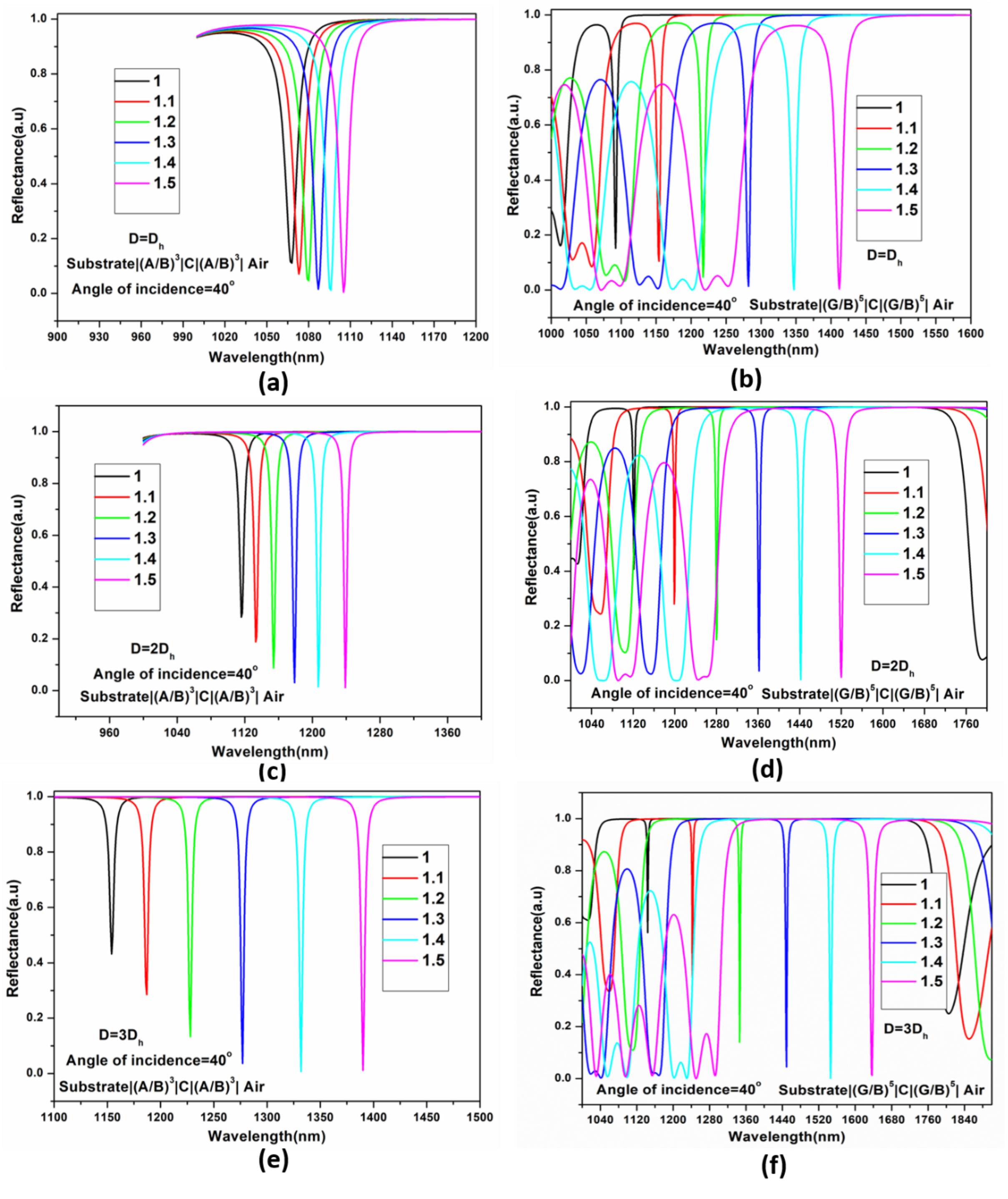

Fig. S3: Effect of varying defect layer thicknesses on sensitivity for the structure **Substrate/(A/B)^3 /C /(A/B)^3 /Air**, (a) D = D_h, (c) D = $2D_h$, (e)D =$3D_h$ And for **Substrate/(G/B)^5 /C /(G/B)^5 /Air** (b) D = D_h, (d) D = $2D_h$, and (f) D = $3D_h$. With incidence angle 40 degrees
